# A combination of kissing molars, maxillary bilateral supernumerary teeth and macrodontia: a rare case report

**DOI:** 10.1186/s12903-020-01092-9

**Published:** 2020-04-16

**Authors:** An Lao, Siyuan Bi, Haoran Cheng, Tiehan Lai, Shengbin Huang, Shufan Zhao

**Affiliations:** 1 Department of Prosthodontics, School and Hospital of Stomatology, Wenzhou Medical University, Wenzhou, People’s Republic of China; 2 Institute of Stomatology, School and Hospital of Stomatology, Wenzhou Medical University, No. 373, Xueyuan West Road, Lucheng District, Wenzhou, 325027 People’s Republic of China; 3 Sunshine Dental Clinic, No. 91, Guyun Road, Gongshu District, Hangzhou, 310000 People’s Republic of China; 4 Department of Oral Maxillofacial Surgery, School and Hospital of Stomatology, Wenzhou Medical University, No. 373, Xueyuan West Road, Lucheng District, Wenzhou, 325027 People’s Republic of China

**Keywords:** Supernumerary teeth, Kissing molars, Bilateral distomolars, Macrodontia

## Abstract

**Background:**

Supernumerary teeth (ST) is defined as an additional number of teeth compared to the normal dental formula. The prevalence rate of ST varies from 0.5 to 3.8% in the permanent dentition. When ST located distal to the third molar is acclaimed as distomolar. Moreover, kissing molar is an extremely scarce condition of distomolars, pointed in the opposite direction in a single follicular space. Meanwhile, macrodontia is also a rare shape anomaly characterized by a large crown and tapering root.

**Case presentation:**

A 22-year-old Chinese man presented a combination of kissing molars, maxillary bilateral supernumerary teeth and macrodontia. Radiographically, two maxillary bilateral distomolars located at the buccal side of adjacent third molars. One mandibular distomolar with the adjacent third molar was contacted by occlusal surfaces while roots were pointed oppositely, which could be diagnosed as KM. Furthermore, the left mandibular third molar can be inferred to be a macrodontia, characterized by a large crown and tapering root. After a thorough investigation, we excluded the possibilities of systemic diseases and genetic inheritance. However, the etiology of this rare combination deserves to be further explored.

**Conclusion:**

The combination of kissing molars, maxillary bilateral supernumerary teeth and macrodontia is very rare, especially presented in the patient with no syndromes. As there were no complications with these conditions, long-term observation has been recommended for the patient. In addition, the true etiology need a further exploration.

## Background

Supernumerary teeth (ST) is defined as an additional number of teeth compared to the normal dental formula [1]. As reported, the prevalence rate of ST varies from 0.3 to 0.8% in the primary dentition and 0.5 to 3.8% in the permanent dentition [1]. Single ST occurs in 76–86% of cases while the prevalence rate of multiple ST (more than three) is less than 1%. Multiple ST is usually accompanied with syndromes or developmental anomalies, such as Gardner syndrome, and cleidocranial dysostosis [2]. In addition, ST occurs in any region of the dentition with a particular predilection for the maxillary anterior area and mandibular premolar area [1]. The extra teeth can be present in a unilateral or bilateral way, as erupted or impacted, as a normal or abnormal shape like tuberculate, conical, supplemental, and odontomes [3]. Interestingly, ST’s occurrence appears to be a gender difference and it is more usual in males than females.

Distomolar, a rare type of ST, is located distal to the third molar. It usually appears to be unilateral, conical and smaller than the third molar. Moreover, few cases have been reported about the bilateral presence of distomolars. Meanwhile, kissing molar (KM) is a scarce condition of distomolars, pointed in the opposite direction in a single follicular space; it consists exactly in full impacted permanent molars which occur only in the lower jaw [4].

Macrodontia is also a rare shape anomaly that has been described as dental gigantism. It is characterized by a large crown, and tapering root. And sometimes macrodontia of premolars may be confused with gemination of adjacent teeth or fusion while these are not the same thing. The prevalence of macrodont permanent teeth is 0.03–1.9%. And there is a higher frequency in males. The large crown size causes problems with teeth eruption and disrupts the dentition [5].

ST always causes various complications. The common choice for clinical treatment on ST is extraction with subsequent therapy like orthodontics [6]. Therefore, the ST is always diagnosed when the patients present with complications.

Hence, we are describing a rare case with a combination of bilateral distomolars, KMs and macrodontia, which has not been reported till date. In addition, we have reviewed the existing literature to focus on the incidence, prevalence, proposed hypothesis for etiology, as well as the management of ST in this report.

## Case presentation

**Fig. 1:**
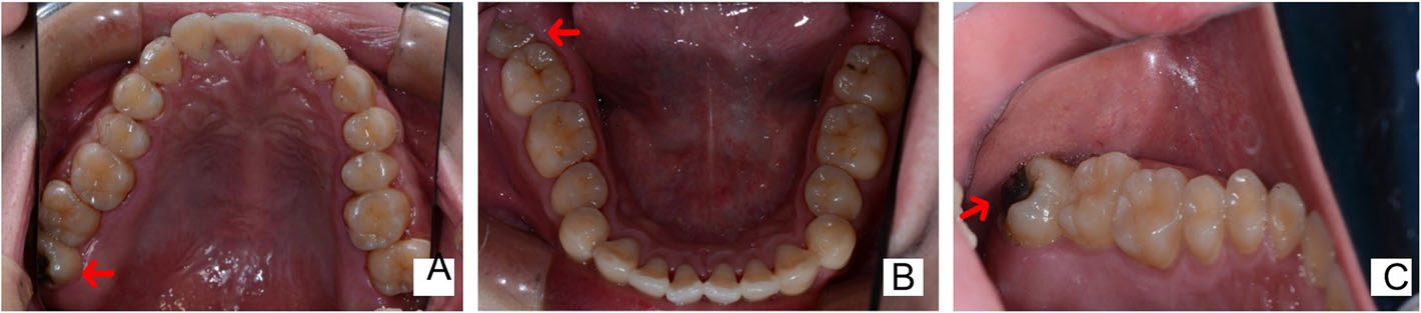
Pretreatment photographs of intraoral examination. (**a** arrowhead) A photo of maxillary dentition showed tooth18 partly erupted. (**b** arrowhead) A photo of mandibular dentition showed tooth38 partly erupted. (**c** arrowhead) The intraoral picture of tooth18 showed it had caries

A 22-year-old man was referred to the Department of Oral Maxillofacial Surgery, School and Hospital of Stomatology, Wenzhou Medical University, China. The patient’s primary complaint was spontaneous pain of right upper posterior teeth for 4 days. On the detailed intraoral examination, we found that the patient’s dentition was normal without abnormal occlusion. Furthermore, Teeth 18 and 38 were partly erupted. And tooth 18 had caries, which have already reached the medullary cavity according to clinical examination. There was no abscess or fistula in the buccal or lingual mucosa and no painful reaction with regard to percussion. So deep caries causing irreversible pulpitis may be the reason why the patient felt pain (Fig. 1a-c).

**Fig. 2:**
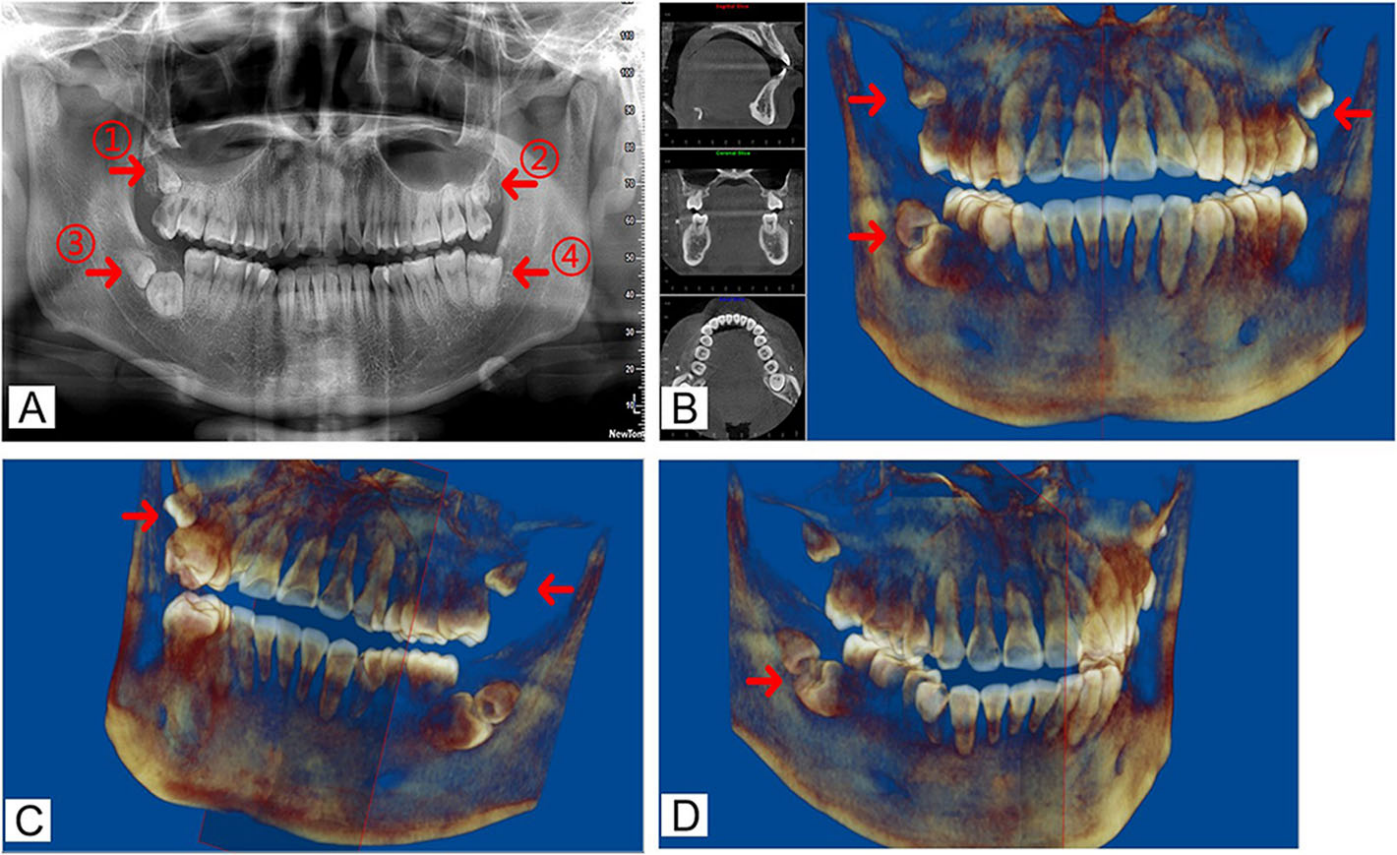
Pretreatment panoramic radiograph and three-dimensional (3D) reconstruction performed by CBCT along with Dolphin software analysis. **a** Dental panoramic tomogram revealed three ST in all four quadrants. Arrowhead ① and ② manifested two ST were located at the root tip of the third molar; arrowhead ③ presented ST was relatively close to the adjacent third molar, and the orientation of them was odd; arrowhead ④ showed the third molar had square shape of crown, extremely short root. (**b** arrowhead) The 3D reconstruction more clearly revealed positions of ST with adjacent teeth. (**c** arrowhead) The 3D reconstruction proved that two distomolars were both grown to the third molar buccally. (**d** arrowhead) The 3D reconstruction presented occlusal surfaces of KMs contacted each other while their roots were pointed in the opposite direction

Then the patient was asked to take a dental panoramic tomogram to examine the condition of dentition more clearly. The results showed that dental caries has already approached the distopulpal horn and there was no low-density shadow around the apical root, which could determine that irreversible pulpitis caused pain symptom and almost exclude the possibility of periapical disease. In addition, the patient was found to have three ST. Two ST in the maxilla were located at the root tip of the third molar, obstructing the natural eruption of third molar (Fig. 2a ① and ②). The position of the third ST was uncommon with its adjacent molar (Fig. 2a ③). Moreover, the crown shape of tooth 38 was square and its size was bigger than normal teeth. Meanwhile, this tooth had an extremely short root, and the root divergent position was close to its root apex (Fig. 2a ④). Radiographic findings revealed macrodont mandibular third molar with its distinct morphological appearance, characterized by a large crown, and tapering root. It can be inferred that the tooth may be macrodontia [7].

ST was found all skeletally ambushed. In order to comprehensively detect positions and morphologies of ST, cone-beam CT (CBCT) detection along with Dolphin software analysis was also performed (Fig. 2b). The bilateral ST was both located at the distal and buccal side of the maxillary third molar (Fig. 2c). ST and the adjacent third molar in the right mandible were contacted each other by occlusal surfaces while roots were pointed in the opposite direction (Fig. 2d). It could be diagnosed that the condition was KM according to the previous study [4].

In order to explore the etiological agent, we collected photos of the patient’s face. Craniofacial development examination showed the patient’s face is symmetrical on frontal view. On the lateral view, the patient showed a straight profile. The lateral X-ray picture showed no frontal sinus, maxillary sinus and papillary dysplasia, cranial suture broadening. It’s indicated that the possibility of cleidocranial dysplasia syndrome was low [8]. Meanwhile, in order to test the genetic possibility, we took dental panoramic tomograms of the patient’s parents and no abnormalities were found. Moreover, family and medical history did not reveal any related positive findings on genetic factors.

## Discussion & conclusion

In this case, the patient exhibited not only a scarce example of multiple ST but also a rare combination of bilateral distomolars, KM-type ST and macrodontia. This kind of case hasn’t been founded till date. ST might occur singly or multiply, unilaterally or bilaterally in any position of the dentition or even in the jaw. The most common type is single, located in the anterior maxilla. A few multiple ST are reported to be accompanied by systematic disorders. Therefore, the presence of three distomolars that we presented, with no syndromes and family history is rare. Especially we represented two peculiar dentition conditions in individuals.

The prevalence rate of distomolars was between 1 and 2.2%. Previous studies suggested that the probability of distomolar occurrence in the maxilla is between 69 and 91% [9]. Interestingly, our case not only showed maxillary and mandibular distomolars, but also appeared bilateral distomolars. Elif Kaya et al. [9] at 2015 reported only three cases of bilateral maxillary distomolars in 10,111 patients aged 18–60 years old. Concerning our case, it is infrequent to find bilateral maxillary distomolars. In addition, our case also represented a more unusual condition-KM. KM is a rare form of inclusions that we only found 32 cases published from 1973 till date. This condition can be found between the second and third molars, and also exited between the third molar and distomolar. Other than cases reported before represent only the occurrences of KM. Our case showed a typical feature of KM, an unwonted combination of bilateral distomolars and macrodontia. Macrodontia is a scarce anomaly of tooth’s shape. Also, macrodontia is mostly reported in mandibular premolars or molars. It is not usual that macrodontia occurred in the third molar like our case [10].

Nowadays the etiology of ST still is not clear. There are many theories trying to explain the occurrence of ST. The tooth germ dichotomy theory proposed that the dichotomy of dental buds leads to the occurrence. While the hyperactivity theory revealed that ST was caused by local, conditional and independent hyperactivity of the dental lamina. In addition, it’s also stated that ST may be hereditary, however, there is no simple Mendelian pattern [11]. And KM is kind of special ST so the theories of occurrence are alike. Some reports proposed high cystic formation or fourth molar may lead to bone loss and finally the existence of KM [12]. Furthermore, macrodontia is usually accompanied by syndromes while the mechanism of macrodontia still is not clear [5]. In this case, parents didn’t have ST or KM. Thus, the probability of heredity was relatively low. Meanwhile, the maxillofacial examination of the patient did not show any maxillofacial abnormalities and cranial clavicular syndrome. Combined with past history, we excluded the possibility of systemic diseases. Therefore, the reason of combination of bilateral distomolars, KM-type ST and macrodontia needs to be further explored.

An accurate diagnosis for ST could be through clinical or radiographic examination. A radiographic examination is needed if abnormal clinical symptoms are found [13]. Nowadays appropriate diagnosis with CBCT is more recommended. As CBCT could provide 3D information of teeth’ morphologies and positions, it is routinely considered to evaluate teeth with volumetric analysis of pulp/tooth ratio [14]. In this case, we performed CBCT detection along with Dolphin software analysis. We confirmed that two ST in the maxillary were located at the buccal side of third molars; ST in the mandible was contacted the adjacent tooth by their occlusal planes, and their roots were pointed in the opposite direction. Compared to the conventional radiographs as radiographic examination, evaluation with the CBCT revealed detailed imaging of significant anatomical structures and objects of interest, with highly accurate anatomical and morphologic imaging in contrast to the intraoperative findings [12].

ST causes many complications, such as root absorption, decayed teeth, ectopic eruption, overcrowding, periapical absorption of permanent teeth, and migration into the nasal cavity or maxillary sinus. The large crown size of macrodontia causes problems with the eruption and disrupts the dentition. But in our case, the patient didn’t show any symptoms or pathologic change associated with ST and macrodontia. Though the dominated treatment of ST is extraction, ST along with the condition of the circumambient teeth should be taken into consideration. While KMs would be treated if they cause harmful symptoms, such as high risk of odontoma, decayed teeth, periodontal complications, cystic pathology or progressive bone loss [15]. Surgical extraction of KMs is the most frequent protocol. Some also presented orthodontic treatment. As for macrodontia, following surgical removal, orthodontic therapy is initiated to correct the malocclusion. In our case, the patient removed tooth 18 to prevent more serious caries. Since there’s no risk of compression and absorption of adjacent roots in the patient’s ST and macrodontia, observation should be reserved for the time being. While it is necessary to take examinations regularly for review, aiming to evaluate development of any related pathologies in the future.

In conclusion, we presented a rare case of the combination of kissing molars, maxillary bilateral supernumerary teeth and macrodontia, which has not been reported till date. However, further investigations about etiology and treatment are still needed to be explored.

## Data Availability

The datasets used and analyzed during the current study are available from the corresponding author on reasonable request.

## Abbreviations

ST: Supernumerary teeth
KM: Kissing molar
CBCT: Cone-beam CT
